# NuMA is required for proper spindle assembly and chromosome alignment in prometaphase

**DOI:** 10.1186/1756-0500-2-64

**Published:** 2009-04-28

**Authors:** Laurence Haren, Nicole Gnadt, Michel Wright, Andreas Merdes

**Affiliations:** 1 Wellcome Trust Centre for Cell Biology, University of Edinburgh, King's Buildings, Edinburgh EH9 3JR, UK; 2 Centre National de la Recherche Scientifique/Pierre Fabre UMR 2587, 3 rue des Satellites, 31400 Toulouse, France

## Abstract

**Background:**

NuMA is a protein that has been previously shown to play a role in focusing microtubules at the mitotic spindle poles. However, most previous work relies on experimental methods that might cause dominant side effects on spindle formation, such as microinjection of antibodies, overexpression of mutant protein, or immunodepletion of NuMA-containing protein complexes.

**Findings:**

To circumvent these technical problems, we performed siRNA experiments in which we depleted the majority of NuMA in human cultured cells. Depleted mitotic cells show a prolonged duration of prometaphase, with spindle pole defects and with unattached, unaligned chromosomes.

**Conclusion:**

Our data confirm that NuMA is important for spindle pole formation, and for cohesion of centrosome-derived microtubules with the bulk of spindle microtubules. Our findings of NuMA-dependent defects in chromosome alignment suggest that NuMA is involved in stabilizing kinetochore fibres.

## Findings

NuMA, the protein of the 'Nucleus and Mitotic Apparatus', is a structural protein in vertebrates of approximately 230 kDa. It localizes to the nucleus during interphase, and accumulates at the spindle poles during mitosis [1]. NuMA has been implicated in the formation of the mitotic spindle, in particular in focusing the spindle poles [2]. Moreover, in recent years it has been shown that part of NuMA localizes to the cell cortex during mitosis where it interacts with the protein LGN/pins [3,4]. It has been suggested that cortical NuMA participates in spindle orientation, a role that has also been attributed to related proteins in Drosophila and Caenorhabditis elegans, termed Mud or LIN-5, respectively [4-9]. So far, the majority of experiments that tested the role of vertebrate NuMA relied on methods such as antibody microinjection, overexpression of NuMA mutants, or depletion of NuMA from cytoplasmic extracts [10-22]. The cumulative evidence from these experiments pointed towards a function of NuMA in crosslinking microtubules at the spindle poles, enabling the formation and maintenance of the bipolar spindle apparatus. The shortcomings of these experiments were that they could not distinguish between a direct effect on NuMA function, and an indirect effect on interacting proteins: 1) Antibodies are large proteins; therefore, upon microinjection they might sterically hinder the function of neighbouring proteins that are in close contact with NuMA. Moreover, antibodies of the immunoglobulin G type may induce crosslinking of NuMA and produce dominant effects that are unrelated to the normal function of NuMA. 2) Similarly, overexpression of mutant forms of NuMA might produce dominant effects due to unphysiological behaviour of the mutant protein, or due to protein aggregates resulting from the overexpression itself. 3) Although depletion of NuMA from cytoplasmic extracts that form spindles or microtubule asters in vitro seems an interesting experimental alternative, it can be disputed as to how closely these assays reflect the mechanisms of real mitosis in a living cell. Furthermore, it cannot be excluded that during depletion of NuMA, interacting proteins are co-depleted that are themselves essential for regular mitosis. Interestingly, a very recent report documented the properties of a loss-of-function allele of NuMA in mouse cells [23]. This mutant form of NuMA lacked exon 22 and was therefore thought to lack binding to spindle microtubules in mitotic cells. Cells expressing this mutant allele and lacking full-length NuMA displayed spindle pole defects, and showed defects in metaphase chromosome alignment [23]. However, it can't be excluded that the mutant allele produced dominant effects, as already discussed for other NuMA mutants.

**Figure 1:**
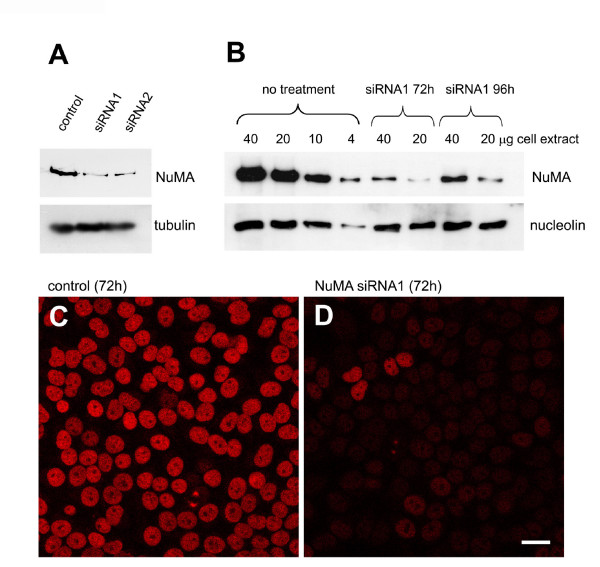
**Depletion of NuMA from HeLa cells by siRNA**. (A) HeLa cells were treated with control dsRNA, or two specific siRNAs against NuMA (siRNA 1 and 2; see main text for description of targeting sequences). Cells were lysed after 72 hours, and lysates were analysed by immunoblot, using antibodies against NuMA (monoclonal antibody NA09L, Calbiochem) or against alpha-tubulin (monoclonal antibody DM1a, Sigma-Aldrich). (B) Immunoblots of decreasing amounts of HeLa lysates from control cells, or cells treated with NuMA siRNA1 for 72 or 96 hours, respectively. Amounts were loaded as indicated. The blots were probed with antibodies against NuMA, or nucleolin as a loading control. Depletion efficiency was determined by scanning of the blots and comparing the intensity of NuMA from depletion experiments to the dilution series of control lysate. (C) Testing of the depletion efficiency of NuMA by immunofluorescence. Left, cells treated with control RNA for 72 hours; right, cells treated with NuMA siRNA1. Bar, 20 μm.

**Figure 2:**
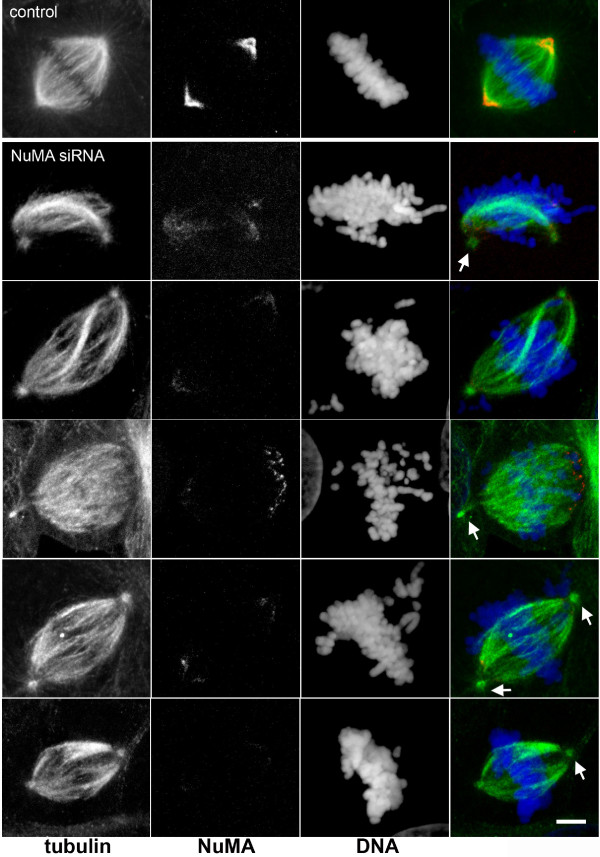
**Unaligned chromosomes and spindle abnormalities in NuMA-depleted cells**. Depletion of NuMA was performed with siRNA2, as described in Fig. 1. Immunofluorescence of microtubules (green) and NuMA (red) was performed using monoclonal anti-NuMA, combined with a rabbit antibody against tubulin. DNA was stained with 4',6-diamidino-2-phenylindole (DAPI, blue). Arrows depict centrosomes that are slightly disconnected from the main body of the spindle in NuMA-depleted cells. The top row shows a cell treated with control RNA, for comparison. Micrographs were taken with a Leica TCS SP confocal microscope, equipped with a PlanApo 100x/1.4NA objective lens (Leica Microsystems). Bar, 5 μm.

**Figure 3:**
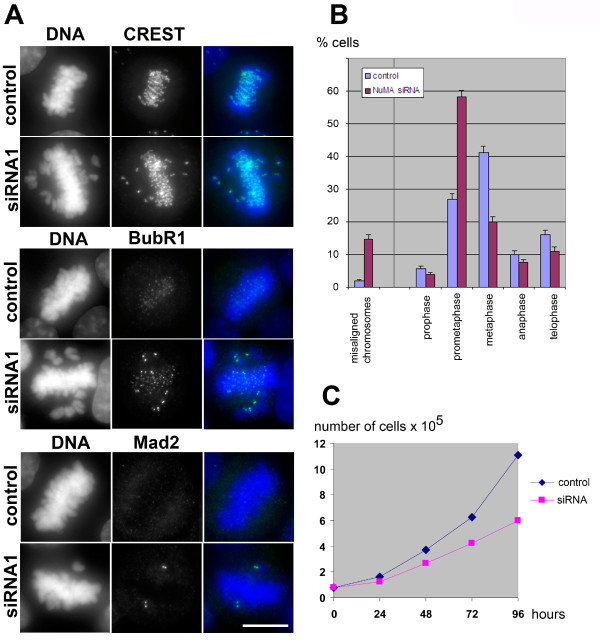
**Silencing of NuMA activates the spindle assembly checkpoint**. (A) Cells treated with NuMA siRNA1 and control cells were processed for immunofluorescence of centromeres with human CREST autoimmune serum (top, green), or with specific antibodies against the checkpoint proteins BubR1 (middle, green), and Mad2 (bottom, green). Antibody against BubR1 was provided by Dr Tim Yen (Fox Chase Center, Philadelphia, PA); antibody against Mad2 was from Berkeley Antibody Company. DNA was stained with DAPI (blue). Bar, 10 μm. (B) The percentage of mitotic cells in different phases is indicated for controls (blue) and cultures treated with NuMA siRNA1 for 72 hours (red). Standard deviations were calculated from n = 706 mitotic control cells, and from n = 601 depleted mitotic cells (data obtained in three independent experiments). The percentages of those prometaphases that show unaligned chromosomes besides mostly aligned chromosomes (as seen in A) are indicated separately on the left. (C) Growth curve of HeLa cells subjected to control treatment or NuMA siRNA1.

Several reports have taken advantage of silencing NuMA expression with siRNA [4,24-28]. In these reports, NuMA was found to be important for cell survival, and in mitosis it was found to interact with tankyrase 1 and to contribute to the formation of multipolar spindles in cancer cells [4,24-26]. However, the potential importance of NuMA in spindle assembly was not clarified. To test this, we performed RNA silencing of NuMA in HeLa cells. Using siRNA oligomers against two different target sequences, we depleted 85 to 90% of NuMA with each oligomer (Fig. 1). The double-stranded siRNA oligomers had the following target sequences: control, CGTACGCGGAATACTTCGA (corresponding to luciferase, [29]); NuMA siRNA1, GGCGTGGCAGGAGAAGTTC [29]; NuMA siRNA2, CTAGCTGAGCTCCATGCCA. The depletion was well visible by immunofluorescence of interphase nuclei (Fig. 1), whereas in mitotic cells some remaining protein was still detectable at the spindle poles. Photometric measurements of NuMA immunofluorescence indicated that the intensity at the poles decreased to 21 to 45% in depleted cells (n = 24). Previous measurements of GFP-NuMA in living cells had revealed that the spindle poles occupy approximately 8% of the volume of the mitotic cell, but concentrate 25% of NuMA, whereby the remaining NuMA is diffusely distributed in the cytoplasm ([30], and unpublished observation). Because the cytoplasmic pool of NuMA was nearly invisible after siRNA, we concluded that almost all of NuMA that resisted depletion concentrated at the poles. With this assumption, a reduction of total NuMA levels to 10% would correspond to an intensity of 40% at the poles, thus matching well our measurements. Most depleted cells were able to form bipolar spindles, but we often observed 'immature' spindles that resembled early stages of prometaphase, occasionally containing unfocused spindle poles (Fig. 2). A small percentage of mitotic cells with supernumerary spindle poles was also seen. In many depleted cells, the centrosome seemed disconnected from the main body of the spindle, and spindle microtubules appeared slightly twisted (Fig. 2). An overall increase of prometaphases was seen among mitotic cells in depleted cultures (27% in controls, compared to 58% in depleted cells, Fig. 3). A strikingly high number of these prometaphases showed chromosomes that were not aligned at the equatorial plate (2% in controls, 15% in treated cells after 72 hours; Fig. 3A, B). The kinetochores of unaligned chromosomes in these cells stained positively for the markers BubR1 and Mad2, indicating that the spindle assembly checkpoint remained active (Fig. 3). Likewise, the small percentage of control cells that contained unaligned chromosomes also stained positively for BubR1 and Mad2 (data not shown). Consistently, cell growth in depleted cell cultures was reduced (Fig. 3C).

**Figure 4:**
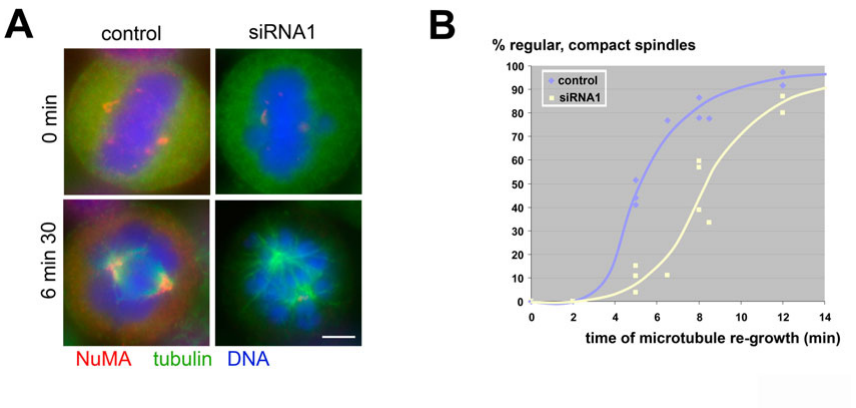
**Spindle microtubule re-growth is delayed in NuMA-depleted cells**. (A) Microtubules were depolymerised on ice for 1.5 hours, and re-polymerised at 37°C. At the indicated time points, cells were fixed and subjected to immunofluorescence of NuMA (red), tubulin (green), and staining of DNA (blue). Spindles in NuMA-depleted cells have a lower microtubule density and are less 'compact'. Bar, 5 μm. (B) Graph, indicating the percentage of regular, compact spindles at the indicated time points of microtubule re-growth (data from four independent experiments; between 50 and 100 cells were counted for each point).

**Figure 5:**
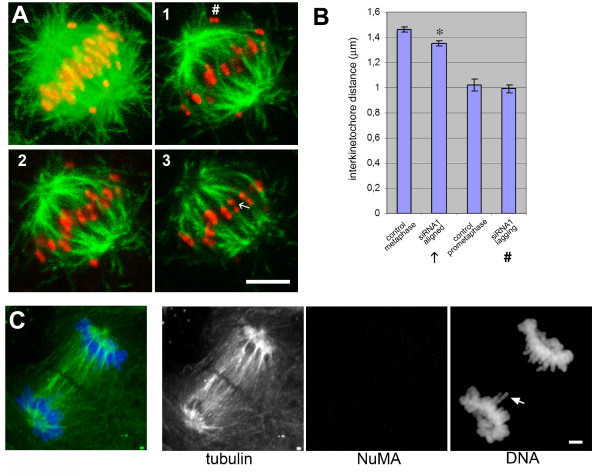
**NuMA depletion induces reduced tension at kinetochores and lagging chromosomes**. (A) Confocal series (frames 1–3) of a NuMA-depleted mitotic cell, showing immunofluorescence staining of kinetochores (using a human CREST autoimmune serum, red), and microtubules (green). On the left, a projection of all sections of the confocal series through the cell is shown. Interkinetochore distances of lagging (#) and aligned (←) chromosomes were quantified and plotted in (B). Error bars represent the SEM (standard error of the mean). Mean values for the aligned chromosomes were significantly different between depleted and control cells (two-tailed τ test, * p < 0.001) (C) NuMA-depleted cell in late anaphase, stained for tubulin (green), NuMA (red), and DNA (blue). Chromosomes are separated to the respective poles, but single lagging chromosomes can be seen, as indicated by the arrow. Bars in A and C, 5 μm.

To determine whether the formation of spindle microtubules was inhibited in depleted cells, we performed assays of microtubule re-growth after cold treatment (Fig. 4). After 6 minutes, we observed four times less spindles in depleted cells than in controls. Besides, many of these spindles that started to assemble in depleted cells showed a lower microtubule density and lacked obvious microtubule bundles such as normally seen in kinetochore fibres (Fig. 4). The full number of spindles was restored after 15 minutes. To determine whether the absence of NuMA had an effect on the tension at kinetochores, we compared the inter-kinetochore distances in control and depleted cells. We found that in depleted cells, those kinetochores that belonged to aligned chromosomes had slightly reduced distances as compared to control cells in metaphase (1.35 versus 1.46 μm; Fig. 5A, B). Unaligned chromosomes in depleted cells had a reduced interkinetochore distance of 0.99 μm, matching the values obtained from early prometaphases in controls (Fig. 5A, B). Unaligned chromosomes were frequently seen to lack amphitelic attachment (Fig. 5A, 1#). Altogether, our results suggest that in depleted cells kinetochores are under reduced tension. Despite such observed defects, NuMA-depleted cells seemed to complete mitosis, as evidenced by the presence of anaphase and telophase spindles (Fig. 5C and Fig. 3B). Occasionally, single lagging chromosomes were seen. This is consistent with our previously published findings [22] and with findings by [23], which indicated that defects due to NuMA inhibition do not interfere with completion of mitosis after the onset of anaphase. Alternatively, it is possible that upon siRNA treatment against NuMA, two different populations of cells are produced: a population in which the vast majority of NuMA is depleted and that shows an arrest in prometaphase, followed either by checkpoint slippage or cell death. A second population, in which significant amounts of NuMA may remain in the cell, may complete mitosis without major errors. Previous studies have shown that silencing of NuMA does indeed lead to apoptosis [24]. Consistently, apoptotic cells were occasionally seen in our cultures after several days of NuMA silencing (data not shown). Because these cells had the tendency to round up and detach from the substratum, we were unable to quantify their percentage by immunofluorescence. So far, we cannot distinguish between the possibility of cell death during mitosis, or cell death after completion of mitosis, eventually as an indirect consequence of improper chromosome segregation.

In conclusion, our depletion experiments suggest that NuMA is necessary for proper spindle formation in prometaphase, and that NuMA-dependent defects manifest in less efficient formation of kinetochore fibres. Improperly formed kinetochore fibres may in turn be responsible for defects in chromosome alignment and tension at kinetochores. Because we observed that even cells that were only partly depleted of NuMA (45% remaining at the mitotic poles) showed defects in chromosome alignment, we favour a model in which reduced levels of NuMA lead to prolongation of prometaphase due to an active spindle assembly checkpoint, until bipolar attachment and tension at kinetochores is finally achieved. We believe that the fraction of NuMA that localizes to the cell cortex in regular cells, as described by [3,4], plays only a minor role in mitotic progress in experiments, since we see significant NuMA accumulation at the cortex only in late phases of mitosis, from metaphase/anaphase onwards, i.e. after chromosome alignment has occurred (unpublished observation).

In our RNA silencing experiments we detected smaller amounts of severely malformed spindles as compared to spindle formation assays after NuMA depletion in Xenopus egg extracts [19]. Drastic spindle defects seen in these extracts included loss of focused poles and prolonged length of the mitotic apparatus [19]. The different results may be explained by a higher sensitivity to experimental manipulation of spindles in vitro compared to spindles in intact cells, or by defects from co-depletion of NuMA-associated proteins, as discussed above, or by the presence of low levels of NuMA remaining at the poles after RNA silencing.

## Competing interests

The authors declare that they have no competing interests.

## Authors' contributions

LH and NG participated in the conception of the work, performed microscopy and biochemical experiments, and contributed to the drafting of the manuscript. MW contributed to the design of the study, drafting of the manuscript, and provided logistic help. AM contributed to conception of the work, performed microscopy experiments, and drafted the manuscript. All authors read and approved the final manuscript.
